# Genetic Variation of the Endangered *Gentiana lutea* L. var. *aurantiaca* (Gentianaceae) in Populations from the Northwest Iberian Peninsula

**DOI:** 10.3390/ijms150610052

**Published:** 2014-06-05

**Authors:** Oscar González-López, Carlos Polanco, Zsuzsanna György, Andrzej Pedryc, Pedro A. Casquero

**Affiliations:** 1 Department of Agrarian Engineering and Sciences, Natural Resources Institute, University of León, Av. Portugal 41, 24071 León, Spain; E-Mail: ogonl@unileon.es; 2 Genetics Lab, Department of Molecular Biology, University of León, Campus de Vegazana, 24071 León, Spain; E-Mail: carlos.polanco@unileon.es; 3 Department of Genetics and Plant Breeding, Corvinus University of Budapest, Ménesi Street, H-1118 Budapest, Hungary; E-Mails: zsuzsanna.gyorgy@uni-corvinus.hu (Z.G.); andrzej.pedryc@uni-corvinus.hu (A.P.)

**Keywords:** alpine plants, *aurantiaca*, Cantabrian Mountains, genetic diversity, geographical isolation, *Gentiana lutea* L., small population, habitat fragmentation, over-exploitation

## Abstract

*Gentiana lutea* L. (*G. lutea* L.) is an endangered plant, patchily distributed along the mountains of Central and Southern Europe. In this study, inter-simple sequence repeat (ISSR) markers were used to investigate the genetic variation in this species within and among populations of *G. lutea* L. var. *aurantiaca* of the Cantabrian Mountains (Northwest Iberian Peninsula). Samples of *G. lutea* L. collected at different locations of the Pyrenees and samples of *G. lutea* L. subsp. *vardjanii* of the Dolomites Alps were also analyzed for comparison. Using nine ISSR primers, 106 bands were generated, and 89.6% of those were polymorphic. The populations from the Northwest Iberian Peninsula were clustered in three different groups, with a significant correlation between genetic and geographic distances. *Gentiana lutea* L. var. *aurantiaca* showed 19.8% private loci and demonstrated a remarkable level of genetic variation, both among populations and within populations; those populations with the highest level of isolation show the lowest genetic variation within populations. The low number of individuals, as well as the observed genetic structure of the analyzed populations makes it necessary to protect them to ensure their survival before they are too small to persist naturally.

## 1. Introduction

*Gentiana lutea* L. (Gentianaceae) is an herbaceous perennial plant native to the mountains of Central and Southern Europe, where their typical habitat is cattle grazing pastures [1]. In summer, the plant produces one tall inflorescence (up to 200 cm) that carries four to 10 pairs of pseudo-umbels, each consisting of 20 flowers, spaced at 5–10-cm intervals. *Gentiana lutea* is self-incompatible and, thus, depends on pollination by insects to produce any seeds, being pollinated mainly by Hymenoptera and Diptera, although neither has special features to facilitate dispersal [2].

Gentian roots are widely used in bitter beverages, in food products and also in traditional medicine to stimulate the appetite and improve digestion [2]. Such uses have generated a great demand, so that more than 1500 tons of gentian root is produced from 6000 tons of the wild stocks every year [3]. The increasing demand has raised concerns about the species’ extinction, and for this reason, gentian is protected by law throughout Europe; but there are divergent regulations across Europe, and the degree of protection varies regionally, concentrating collections where the regulation is laxer.

*Gentiana lutea* L. includes *G. lutea* var. *aurantiaca* (M. Lainz), *G. lutea* L. subsp. *vardjanii* Wraber and *G. lutea* L. subsp. *montserratii* (Vivant ex Greuter) Romo. The populations of the Iberian Peninsula correspond to *G. lutea*, except for small areas of the Pre-Pyrenees and Central Pyrenees, where *G. lutea* L. subsp. *montserratii* is endemic. However, most of the populations from the Northwest Iberian Peninsula have flowers ranging in color from orange to almost red, as compared to the yellow flowers of *G. lutea.* These populations have been classified as *Gentiana lutea* L. var. *aurantiaca* [4].

The Iberian Peninsula is the northwestern region of the Mediterranean Basin hotspot, one of the world’s biodiversity hotspots, with a high diversity in endemic vascular plant species. As Europe’s vacation destination, populations of threatened species are increasingly fragmented and isolated to make way for resort development and infrastructure.

This study mainly focuses on the wild populations of *G. lutea* located in northwestern León province (Spain), in the Cantabrian Mountains. In this region, gentian has been collected historically, for sale and use in medicinal remedies, as in other European regions. Due to the slow growth of this species, its populations have been decimated, and now, they are close to their disappearance and/or are difficult to recover. The main economic sources in the area include coal mining (ceasing activity), ranching and tourism, which all also directly affect gentian populations. People in this area have begun to collect gentian again in a furtive way for extra income due to the current economic crisis. Besides, the local tradition of burning the mountain hillsides to create new pastures for free-range cattle farming can affect the gentian populations, because even though mature plants tolerate fire, undeveloped young plants die because of burning or drying.

Understanding the level of genetic diversity and the population’s genetic structure is important for endangered plant species, because this allows the establishment of effective and efficient conservation practices and can guide choices for their genetic management. Although there are different botanical [5,6] and chemical [7] studies of *G. lutea*, the genetic variability at population levels remains unknown for this species to date. Nowadays, it is possible to use several molecular methods to analyze the genetic variability in plant species. One of them, inter-simple sequence repeat polymorphism (ISSR), has been successfully used for genetic analysis in the case of medicinal plants, requiring no prior knowledge of the DNA sequence and being universally applicable as dominant markers [8] for rapid exploratory work on new species. Furthermore, ISSRs have been demonstrated to be useful for the analysis of inter- or/and intra-specific genetic diversity in different Gentianaceae species [9,10,11,12].

The aim of this study was to investigate for the first time the level of genetic diversity, within and among populations of *G. lutea* L. var. *aurantiaca* from the northwest of the Iberian Peninsula using ISSR markers and to compare their relationship and variation with other populations of *G. lutea* and *G. lutea* L. subs. *vardjanii* collected in the Pyrenees and Dolomite mountains, respectively. The knowledge of the genetic diversity and variation within and among the populations not only enhances our understanding of population dynamics, adaptation and evolution, but also provides useful information for biological conservation of these endangered species*.*

## 2. Results

The amplification of the ISSR fragments in the 123 individuals, analyzed with nine primers, yielded 106 unambiguous and reproducible electrophoretic bands ranging from five to 17 bands for each of the primers, with an average of 11.7 bands per primer. Ninety-five bands (89.6%) were polymorphic when comparing all the samples (Table 1); of the 106 bands, 43 (43.4%) were found in the three geographical regions studied, and 21 (19.8%), three (2.8%) and 12 (11.3%) bands were detected only at the Cantabrian (*G. lutea* L. var. *aurantiaca*), Pyrenees (*G. lutea*) and Dolomite Mountains (*G. lutea* L. subsp. *vardjanii*), respectively. Among populations of the variety, *aurantiaca*, the wild populations, CMV, CVJ and CTL, and the cultivated population, FCP, showed just one, two, one and three private loci, respectively. AMOVA analysis revealed significant (*p* < 0.001) genetic differences among the three taxonomical units analyzed (Table 2). Of the total genetic diversity, 57% was attributable to among taxons and the remaining 43% to within taxons. The percentages of polymorphic loci for a single population ranged from 18.77% (CTL) to 38.68% (CVJ) with an average of 27.79% in the Cantabrian Mountains populations. The Dolomite Mountain populations showed a significant lower mean value (18.16% at *p* = 0.05 according to the Student’s *t*-test), although the different number of samples analyzed for each population may be responsible for such results. The average gene diversity was estimated to be 0.0900 at the population level and 0.2168 at the species level. The CVJ population showed the highest level of genetic diversity (0.1329), while the DBT population exhibited the lowest (0.0546). The average Shannon’s indices showed a strong correlation (Pearson’s *r* = 0.999) with gene diversity, and they were 0.1342 at the population level and 0.3415 at the species level, respectively. The values of gene diversity and Shannon’s index showed a similar trend to the percentages of polymorphic loci.

**Table 1:** Genetic variation in populations of *Gentiana lutea* (*G. lutea*) detected by inter-simple sequence repeat (ISSR) markers.

Geographic Region (Taxon)	Population Names	*N*	*n*	PPL	*N*_a_	*N*_e_	*I*	*H*_e_ (S.E.)
Cantabrian Mountains (*G. lutea* var. *aurantiaca*)	CMV	8	33	31.13	1.3113	1.1743	0.1579	0.1042 (0.0167)
	CLU	8	26	24.53	1.2453	1.1297	0.1192	0.0780 (0.0149)
	CTB	8	32	30.19	1.3019	1.1800	0.1568	0.1046 (0.0172)
	CSN	8	27	25.47	1.2547	1.1361	0.1218	0.0801 (0.0154)
	CLT	8	29	27.36	1.2736	1.1530	0.1398	0.0922 (0.0159)
	CPN	8	35	33.02	1.3302	1.2033	0.1716	0.1152 (0.0181)
	CVJ	8	41	38.68	1.3868	1.2266	0.1993	0.1329 (0.0184)
	CTN	8	32	30.19	1.3019	1.1878	0.1608	0.1081 (0.0176)
	CTR	8	21	19.81	1.1981	1.1264	0.1066	0.0719 (0.0153)
	CTL	8	20	18.87	1.1887	1.1290	0.1082	0.0736 (0.0155)
	FCP	8	28	26.42	1.2642	1.1583	0.1363	0.0911 (0.0165)
	Mean values		29.45	27.79	1.2779	1.1640	0.1435	0.0956 (0.0058)
	Group	88	70	66.04	1.6604	1.2346	0.2361	0.1480 (0.0028)
Pyrenees (*G. lutea*), Dolomite Alps (*G. lutea* L. sub sp. *vardjanii*)	Group	15	30	28.30	1.283	1.1883	0.1581	0.1074 (0.0179)
	DBN	5	18	16.98	1.1698	1.1138	0.0964	0.0654 (0.0147)
	DPL	5	24	22.64	1.2264	1.1502	0.1263	0.0856 (0.0164)
	DTZ	5	20	18.87	1.1887	1.1304	0.1078	0.0735 (0.0157)
	DBT	5	15	14.15	1.1415	1.0978	0.08	0.0546 (0.0139)
	Mean values		19.25	18.16	1.1816	1.1231	0.1026	0.0698 (0.0065)
All	Group	20	31	29.20	1.2925	1.1735	0.1499	0.1002 (0.0017)
	Global mean		26.9	25.40	1.2541	1.1553	0.1342	0.0900 (0.0052)
	Total	123	95	89.60	1.8962	1.3446	0.3415	0.2168 (0.0166)

*N*, sample size; *n*, number of polymorphic loci; PPL, percentage of polymorphic loci; *N*_a_, observed mean number of alleles per locus; *N*_e_, effective mean number of alleles per locus; *I*, Shannon’s information index; *H*_e_, Nei’s gene diversity; S.E., standard error.

**Table 2:** Summary of the analysis of molecular variance (AMOVA) using GenAIEx.

Analysis	Source of Variation	d.f.	Sum of Squares	Estimated Variance	% Total Variance	Φ	Significance (*p*)
*G. lutea*	Among taxons	2	552.721	9.786	57	0.568	<0.001
	Within taxons	120	891.962	7.433	43		
*G. lutea* var. *aurantiaca*	Among clusters *	2	123.608	1.765	20	0.198	<0.001
	Among populations	8	124.688	1.208	14	0.169	<0.001
	Within populations	77	456.250	5.925	67	0.334	<0.001

d.f., degree of freedom; * three clusters obtained after analysis by population structure methods.

**Figure 1:**
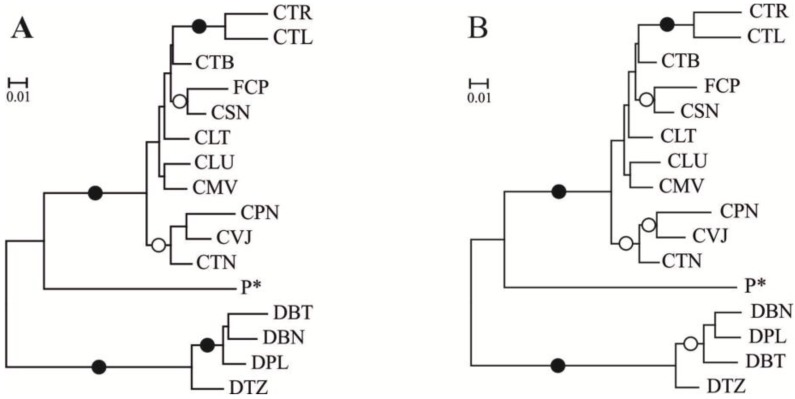
Unrooted neighbor joining tree of *G. lutea* populations obtained using pairwise Nei’s genetic distance (**A**) and the 1 − Q_xy_ distance (**B**). P*, Pyrenees samples grouped. Black circles in nodes indicate bootstrap support ranging from 90% to 100%; White circles in nodes indicate bootstrap support ranging from 70% to 89%.

Pairwise Nei’s genetic distance based on band frequencies ranged from 0.0261 (between the CTB and CLT populations) to 0.3998 (between the CTR and DBN populations), with a mean of 0.1734. The neighbor joining cladogram based on these genetic distances clustered the populations according to their geographical regions of origin (Figure 1A). A nearly identical cladogram was obtained when the genetic distance based on the genetic diversity index for dominant markers, *Q*_xy_, was used (Figure 1B). Two clusters of populations of the variety, *aurantiaca*, from the Cantabrian Mountains, were obtained in both cladograms with high bootstrap values: the cluster formed by CTL and CTR populations, both located in the southwest of the region, and the cluster formed by CPN, CVJ and CTN, the three populations located in the east of the sampled region.

**Figure 2:**
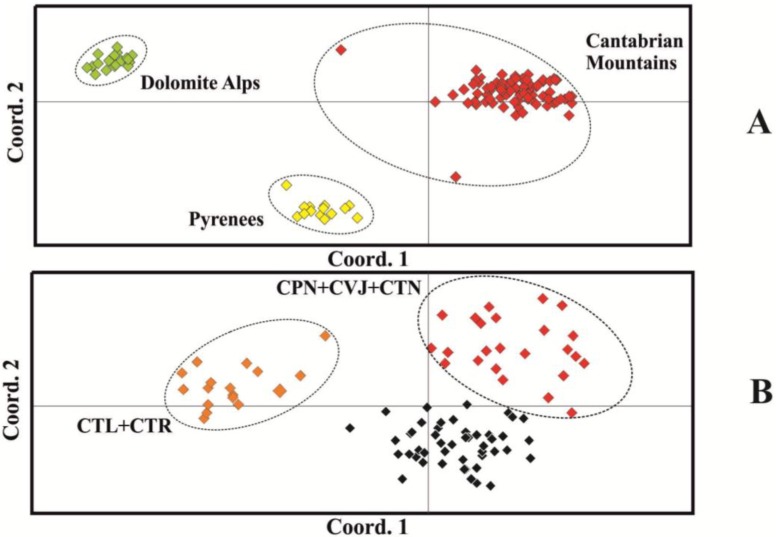
Two-dimensional ordination of principal coordinate (coord.) analysis of 123 individuals of *G. lutea* (**A**) and the subset of 88 individuals of *G. lutea* var. *aurantiaca* from the Cantabrian Mountains (**B**) using Jaccard’s genetic distance between individuals.

In the PCoA (principal coordinates) analysis using Jaccard’s genetic distance matrix for the 123 samples, the first two coordinates that accounted, respectively, for 40.33% and 24.95% of the total genetic variability clearly discriminate the three geographical groups of individuals (Figure 2A). PCoA analysis was also carried out using only the 88 samples of the variety, *aurantiaca*, from the Cantabrian Mountains, showing a clearly separated cluster of individuals from the CTL and CTR populations, with the first two coordinates explaining 29.21% and 22.69% of the variation, respectively. All the individuals from eastern populations (CPN, CVJ and CTN) were also grouped, but without a clear boundary that separates the rest of the individuals (Figure 2B).

**Figure 3:**
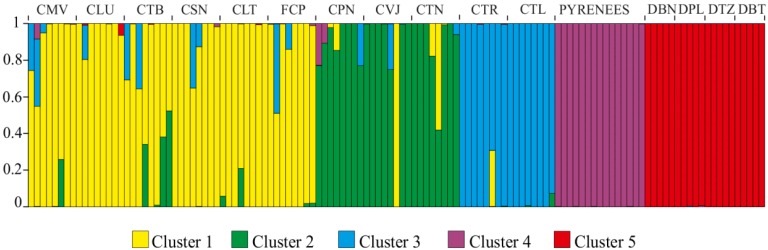
Probability of assignment to each of the five clusters obtained with the Bayesian approach (Structure, version 2.3.4 [13]) for each of the 123 individuals of *G. lutea* analyzed. Individuals are sorted according to their population of origin (codes on top).

The analysis of individual multilocus genotypes of the 123 samples using the Structure algorithm showed the best clustering solution for *K* = 5 (∆*K* = 8.15 with the most closely values of 4.69 and 3.10 for *K* = 4 and *K* = 7, respectively). Figure 3 shows the results obtained after the Bayesian analysis performed for *K* = 5. All the individuals from the Pyrenees and the Dolomite Mountains are clearly separated into two independent clusters, respectively. The 88 individuals from the Cantabrian Mountain populations were assigned to the three remaining clusters: (i) individuals from CTL and CTR (southwestern populations); (ii) individuals from CPN, CVJ (except one individual) and CTN (eastern populations); (iii) individuals from the CMV, CLU, CTB, CSN, FCP and CLT (northwestern populations). Individuals showing probabilities of assignment to more than one cluster were observed in all of the Cantabrian Mountains populations, revealing that there has been some gene flow between clusters. The proportions of individuals that have at least a 5% of probability of assignment to another cluster were 33.3% for the northwestern population group, 37.5% for the eastern population group and 12.5% for the southwestern population group. The *Z*-test (*p* < 0.5, two-tailed) comparing such proportions resulted in a non-statistically significant difference.

AMOVA analysis further revealed significant (*p* < 0.001) differences among the clusters and populations of *G. lutea* var. *aurantiaca*. Of the total genetic diversity, 20% was attributable to among the above clusters of populations, 14% among the populations and the remainder (67%) to within population diversity (Table 2).

The Mantel test detected a significant correlation (*r* = 0.945; *p* = 0.001) between Nei’s distance and geographical distance for the 16 populations (samples from the Pyrenees were grouped as a single population located at PBQ coordinates) and also for the 11 populations from the Cantabrian Mountains (*r* = 0.844; *p* = 0.001) when analyzed independently. No significant correlation (*r* = 0.457; *p* = 0.159) was observed when the four populations from the Dolomite Mountains were analyzed alone. The Mantel test using Jaccard’s distances calculated for the samples from the Pyrenees Mountains did not detect a significant correlation with the geographical distances (*r* = 0.248; *p* = 0.062) between those populations.

## 3. Discussion

There are some publications about gentians, where the use of molecular markers are an important part of the study, like Struwe and Albert [14], with a large study about Gentianaceae; another is more specific in the phylogeny of the European gentians based on the analysis of chloroplast DNA [15]. However, we report the first study of genetic polymorphism in *G. lutea* L. populations using ISSR markers. This is also the first study of variation using samples that correspond to the description of *G. lutea* L. var. *aurantiaca*. Although ISSR markers have not been used in *Gentiana lutea* L. earlier, this methodology has been used successfully in diversity and botanic studies of other gentians [9,10,11,12].

Applying different statistics and analysis (Nei’s distance, *Q*_xy_ genetic diversity, Jaccard’s distance and Structure for multilocus genotypes), we observed clear genetic differences between samples collected in the Dolomite Alps (*G. lutea* L. subsp. *vardjanii*) and those sampled in the Iberian Peninsula (*G. lutea* L. and *G. lutea* L. var. *aurantiaca*), as expected, due to their taxonomical classification. Furthermore, the individuals from the Pyrenees and Cantabrian Mountains are also clearly differentiated in our analysis, giving support to previous taxonomic classifications for the red flowered populations from the Cantabrian Mountains, such as the subspecies, *aurantiaca* [16], or, even, the species, *G. aurantiaca* [17].

Renobales [4] indicated that the orange or reddish corolla was the only significant morphological difference when comparing with yellow gentian (*G. lutea* L.), and therefore, he proposed their classification as a variety. However, we also reported a large number of ISSRs bands (21 out of 106, 19.8%) detected only in the red flowered plants from the Cantabrian Mountains populations. This percentage is about twice as much as the percentages obtained when the gentian species, *G. atuntsiensis* (8.5%) and *G. striolata* (11.3%), were compared using 129 ISSR bands [10].

Renobales [4] stated the existence of the variety, *aurantiaca*, all along the Cantabrian Mountains, but the Ventana Pass (43°03'20.86''N 6°00'28.03''W, approximately in the center of the Cantabrian Mountains) was set as the easternmost point from which their populations are less common, and the plants show this distinguishing feature attenuated. Anchisi [18] also describes a population at Laurentii Lake, in the Pyrenees, showing the same reddish corolla. We reported here the presence of populations that correspond to the description of *G. lutea* L. var. *aurantiaca*, located outside of the growing areas described by Renobales [4] in the Cantabrian Mountains.

The analysis of the Cantabrian Mountain populations revealed that they are clustered in three different groups with a significant correlation between genetic and geographic distances. The analysis of genetic distances using neighbor joining trees and PCoA methods showed a well-defined cluster, including the two southwestern populations, CTL and CTR. The Bayesian analysis of individual genotypes also separated CTL and CTR from the rest of the populations and grouped the eastern populations (CPN, CVJ and CTN) in a different cluster. Besides, AMOVA analysis assigned 20% of the total variance to differences among the three clusters. This population structure can be explained by the lack of continuity in the typical mountainous habitat where gentian grows. This discontinuity might have generated the partial isolation of the CTL and CTR populations. *Gentiana lutea* L. is a self-incompatible species and, thus, depends on pollination by insects to produce any seeds [19,20]. Outcrossing is further suggested by field observations of flowering plants attracting bumblebees, known to be the foremost pollinators of many alpine *Gentiana* species, such as *G. lutea* L. [21] or *G. cruciata* L. [22]. Their long foraging flights may result in relatively frequent long-distance pollen dispersal in *G. lutea*. The small and light seeds can be blown by the wind, and gene flow by seeds can occur among close populations. Therefore, this potential for dispersal by both pollen and seeds may help to explain gene flow between neighboring populations and genetic isolation between widely separated populations.

Zhang *et al.* [10] reported high levels of ISSR diversity in *G. striolata* (percentage of polymorphic loci (PPL) = 80.5% at the species level, with a range from 40.42% to 52.57% at the population level) and *G. atuntsiensis* (PPL = 70.2% at the species level, with a range from 33.41%–46.58% at the population level). Several endangered Chinese gentians (*Gentiana macrophylla* Pall, *Gentiana dahurica* Fisch, *Gentiana straminea* Maxim and *Gentiana crassicaulis* Duthie ex Burk) showed lower levels of ISSR diversity at the species level, with a range of PPL = 43.75%–67.71% [12]. The estimates of genetic diversity in *G. lutea* L. var. *aurantiaca* demonstrated a remarkable level of genetic variation among populations (PPL = 66.04%, Nei’s gene diversity (*H*_e_) = 0.1480) and low-moderate genetic variation within populations when compared with *G. striolata* (PPL for a single population ranged from 18.87% to 38.68%; the average genetic diversity at the population level *H*_e_ = 0.0956). The most threatened taxa have lower genetic diversity than closely related, non-threatened taxa, indicating reduced reproductive fitness and elevated extinction risks [23]. *Gentiana lutea* L. var. *aurantiaca* is a perennial herb with a patchy distribution. The populations are often located in distant mountains and are isolated from each other by plateaus or valleys. Southwestern populations (CTL and CTR, partially isolated from the rest) show the lowest level of genetic diversity (PPL = 18.87 and 19.81; *H*_e_ = 0.0719 and 0.0736), due to the higher genetic isolation of these two populations.

Rare species are typically considered to be genetically less variable than common and widespread species [23]. This indicates that the level of genetic variation might be less strongly associated with population size in rare plants compared with common plants. This is because genetic variation is likely to be low in all populations of rare plants and higher in all populations of common plants regardless of the size of the populations [24]. The observed generality of the positive relationships between population size, plant fitness and genetic diversity implies that the negative effects of habitat fragmentation on plant fitness and genetic variation are common. Moreover, the stronger positive associations were observed in self-incompatible species [25] and, to some degree, in rare species, such as *G. lutea*, that are, to a greater extent, affected by the effects of habitat fragmentation [24]. Kery *et al.* [25] studied reproduction and offspring performance in relation to population size in *G. lutea*. Reproduction was strongly reduced in small populations, where plants produced fewer seeds per fruit and per plant. Reproduction was most strongly depressed in populations consisting of fewer than 500 plants, which occurs in the studied gentian populations (with less than 150 plants per population). These small populations may face an increased short-term risk of extinction, because of reduced reproduction, and an increased long-term risk, because they are less able to respond to environmental changes. There is controversy about the impact of genetic factors on the risk of extinction for threatened species and populations in nature [26]. Population size is reduced by habitat loss, over-exploitation, the impact of introduced species and pollution, until it reaches a point where stochastic factors further elevate extinction risk [27].

The genetic structure of plant populations reflects the interactions of various evolutionary processes, including the long-term evolutionary history, such as shifts in distribution, habitat fragmentation and population isolation, mutation, genetic drift, mating system, gene flow and selection [28]. A high level of population differentiation may be explained by genetic drift and habitat fragmentation, leading to the genetic isolation of populations [29]. Human activity is the main cause of this habitat fragmentation, reduced population size and, consequently, restricted gene flow [30]. The observed genetic structure of the analyzed populations of *Gentiana lutea* L. var. *aurantiaca* from the Cantabrian Mountains implies that as many populations of this variety as possible should be considered for conservation practice.

## 4. Experimental Section

### 4.1. Study Species and Population Sampling

**Figure 4:**
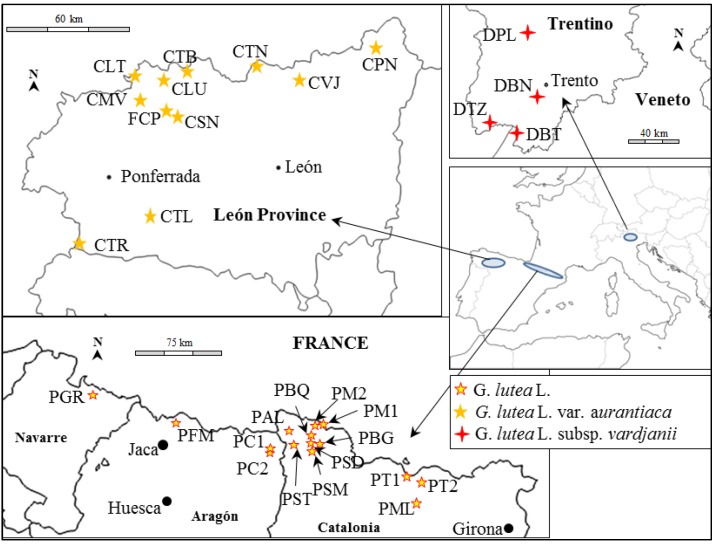
Maps showing the location of sampled populations of *Gentiana lutea* L., *G. lutea* subsp. *vardjanii* and *G. lutea* var. *aurantiaca.* Population names correspond to those given in Table 3.

Root samples of 123 individuals were collected from three regions (Figure 4; Table 3): eight individuals from each of 10 populations of *G. lutea* L. var. *aurantiaca*, in the Cantabrian Mountains (northwest of Iberian Peninsula); one sample of *G. lutea* L. from fifteen locations along the Pyrenees, and five individuals from each of four populations of *G. lutea* subsp. *vardjanii* from the Dolomite Alps, near Trento (Italy). All wild populations studied are endangered due to their small size.

**Table 3:** Populations of *G. lutea* surveyed.

Location	Pop name (Figure 1)	Latitude (North)	Longitude	Altitude (m)	Sample Size
Matalavilla	CMV	42°52'13''	06°23'06''W	1794	8
Lumajo	CLU	42°59'14''	06°15'33''W	1396	8
Torre de Babia	CTB	43°00'47''	06°05'59''W	1801	8
Senra	CSN	42°50'07''	06°11'25''W	1724	8
Leitariegos	CLT	42°59'18''	06°25'26''W	1880	8
Pontón	CPN	43°06'02''	05°00'29''W	1278	8
Valdeteja	CVJ	42°56'28''	05°27'34''W	1419	8
Tonin	CTN	43°00'15''	05°41'00''W	1294	8
Trevinca	CTR	42°09'30''	06°44'29''W	1727	8
Teleno	CTL	42°21'20''	06°25'22''W	1867	8
Cultivated field	FCP	42°52'09''	06°12'49''W	1439	8
Collada de Tosas 1	PT1	42°20'53''	01°59'08''E	1738	1
Collada de Tosas 2	PT2	42°20'29''	02°01'49''E	1654	1
La Molina	PML	42°17'13''	02°02'07''E	1578	1
La Bonaigua	PBG	42°40'10''	00°58'11''E	1813	1
Salardu	PSD	42°40'20''	00°54'58''E	1516	1
Montgarri	PMG	42°45'27''	01°01'58''E	1602	1
Montgarri 2	PMA	42°45'33''	01°01'14''E	1588	1
Baqueira	PBQ	42°43'31''	00°55'59''E	1764	1
Sant Maurici	PSM	42°39'28''	00°55'24''E	1516	1
Artiga de Lin	PAL	42°40'50''	00°42'24''E	1458	1
Senet	PST	42°36'23''	00°46'01''E	1487	1
Cerler 1	PC1	42°33'55''	00°34'01''E	1902	1
Cerler 2	PC2	42°33'43''	00°34'03''E	1892	1
Garaioa	PGR	42°53'23''	01°13'51''W	1170	1
Formigal	PFM	42°47'45''	00°24'11''W	1701	1
Bondone	DBN	46°00'34''	11°02'50''E	1557	5
Peller	DPL	46°19'04''	10°57'34''E	1904	5
Tremalzo	DTZ	45°50'15''	10°41'23''E	1694	5
Brentonico	DBT	45°47'16''	10°54'04''E	1482	5

Eight individuals from a cultivated population of *Gentiana lutea* L. var. *aurantiaca* have also been analyzed. This population was cultivated in an experimental field located in the Cantabrian Mountains where natural conditions allow gentian also to be found growing wild in the area.

Populations were sampled randomly; individuals 5–10 m from each another were chosen to avoid collecting the same plant, since, due to their root development, it is possible that several shoots correspond to the same individual. The altitude of the sampled populations ranged from 1170 to 1904 m. The collected fresh roots were dried in a ventilated oven at 38 °C for 72 h to avoid degradation of their chemical compounds. Dried roots were stored at room temperature with silica gel, since these roots are very hydrophilic.

### 4.2. DNA Extraction and Polymerase Chain Reaction (PCR) Amplification of Inter-Simple Sequence Repeat Polymorphism (ISSR) Markers

Genomic DNA was extracted from 0.2 g of dried root ground using a hammer mill with a 1-mm diameter sieve. The extraction protocol was based on the CTAB (Cetyl Trimethyl Ammonium Bromide) method [31]. DNA concentration and quality was assessed using a NanoDrop spectrophotometer (NanoDrop Technologies, Wilmington, DE, USA) and on 1% agarose gel.

Polymerase chain reaction (PCR) was performed in 15 µL of reaction volume containing 100 ng DNA, 10× DreamTaq Green Buffer (Thermo Scientific, Waltham, MA, USA), 2.5 mM MgCl_2_, 0.02 mM dNTP mix, 5 µmol of primers, 0.25 units of DreamTaq Green DNA Polymerase (Thermo Scientific) and sterile distilled water. Sixteen primers from UBC primer set No.9 (Biotechnology Laboratory, University of British Columbia, Vancouver, BC, Canada), were selected based on previous studies on other Gentianaceae species [9,10,12,32,33] and were screened for PCR amplification. Nine primers (UBC 807, 809, 810, 812, 817, 825, 827, 842 and 857) that gave clear and reproducible banding patterns in *G. lutea* were chosen for the analysis. For the amplification of ISSR fragments, the following program was used: initial denaturation at 95 °C for 1 min; followed by 45 cycles of 94 °C for 30 s, 50 °C for 30 s, 72 °C for 2 min; and a final synthesis at 72 °C for 10 min.

The PCR products were applied on a 1.5% (*w*/*v*) ethidium bromide-stained agarose gel in 1× TBE (Tris–Borate–EDTA) buffer with xylencyanol loading buffer. PCR products were separated for 2 or 3 h (depending on the primer used) at 100 V, avoiding distortions caused by higher voltages. The amplified DNA fragments were documented by using image analysis software Total Lab 1.2 (TotalLab Ltd., Newcastle upon Tyne, UK). The ISSR amplification protocol was reproduced at least twice for each DNA sample.

### 4.3. Data Analysis

Only bands that could be unambiguously scored were used in the analysis. Owing to the dominant character of ISSR markers, each ISSR band was treated as a binary character and was scored as present (1) or absent (0), and it was assumed that each observed band represented the phenotype at a single biallelic locus. Popgene version 1.32 [34] was used to calculate the genetic diversity parameters of the populations: the percentage of polymorphic loci (PPL), the observed number of alleles (*N*_a_), the effective number of alleles (*N*_e_), the gene diversity (*H*_e_) [35] and Shannon’s information index (*I*) [36]. The fifteen samples obtained from different locations across the Pyrenees Mountains were synthetically grouped for comparison purposes.

The population structure was studied using different methods. First, we obtained an unrooted neighbor joining cladogram [37] based on Nei’s genetic distance [38] matrix between populations. The cladogram was constructed using the NEIGHBOR module in PHYLIP version 3.695 [39]. The genetic diversity, *Q*_xy_ [40], matrix between populations was also calculated, and a second dendrogram was produced from the genetic distances (estimated as 1 − *Q*_xy_) as above. The significance of the branch order for both cladograms was examined independently using 100 bootstraps across the set of loci to generate neighbor joining trees. A majority rule consensus tree was constructed by using the CONSENSE module in PHYLIP.

Grouping of the samples was also carried out by PCoA analysis based on Jaccard’s genetic distance matrix of the individuals, and implemented in the software, Genetic Analysis in Excel (GenAIEx) version 6.5 [41].

The third approach used the program, Structure version 2.3.4 [13], which identifies clusters of related individuals from multilocus genotypes. Individuals were assigned (probabilistically) to a cluster or jointly to two or more clusters if their haplotypes indicated that they are admixed; each cluster is characterized by a set of allele frequencies at each locus [13]. To choose the best number of genetic clusters (*K*), multiple values were tested (from 1 to 7) using a length of burning period of 10,000 steps and 10 repetitions. The results were analyzed using the on-line tool, Structure Harvester [42], which implements the method of Evanno, Regnaut and Gaudet [43] to detect the true number of clusters in a non-homogeneous sample of individuals.

Analysis of molecular variance (AMOVA) was also used to describe the genetic structure and variability among and within populations using GenAIEx version 6.5 [41]. The variance components were tested statistically by nonparametric randomization tests using 999 permutations.

In order to test the correlation between genetic and geographic distances among populations, the Mantel test was performed using GenAIEx (version 6.5), computing 999 permutations.

## 5. Conclusions

The gentian populations from the Northwest Iberian Peninsula were clustered in three different groups, with a significant correlation between genetic and geographic distances. *Gentiana lutea* L. var. *aurantiaca* showed a remarkable level of genetic variation, both among populations and within populations; those populations with the highest level of isolation show the lowest genetic variation within populations. The low number of individuals, as well as the observed genetic structure of the analyzed populations makes it necessary to protect them to ensure their survival before they are too small to persist naturally.
